# FIFA 11+ KIDS in the prevention of soccer injuries in children: a systematic review

**DOI:** 10.1186/s13018-024-04876-9

**Published:** 2024-07-18

**Authors:** Ana Paula Ramos, Raphael Schmidt de Mesquita, Filippo Migliorini, Nicola Maffulli, Rodrigo Okubo

**Affiliations:** 1 Departament of Physiotherapy, University of South of Santa Catarina (UNISUL), Florianópolis, SC Brazil; 2 Physical Therapy Graduate Program, Department of Physiotherapy, University of the State of Santa Catarina, Florianópolis, SC Brazil; 3 Department of Orthopedics and Trauma Surgery, Academic Hospital of Bolzano (SABES-ASDAA), Via Lorenz Böhler 5, 39100 Bolzano, Italy; 4 Faculty of Medicine and Psychology, University La Sapienza, Rome, Italy; 5 Faculty of Medicine, School of Pharmacy and Bioengineering, Keele University, Stoke on Trent, ST4 7QB England; 6 Barts and the London School of Medicine and Dentistry, Centre for Sports and Exercise Medicine, Queen Mary University of London, Mile End Hospital, London, E1 4DG England; 7 Department of Life Sciences, Health, and Health Professions, Link Campus University, 00165 Rome, Italy

**Keywords:** FIFA kids, Children, Soccer

## Abstract

**Background:**

The “FIFA 11+” is an injury prevention program conceived for soccer athletes aged over 14. The use of FIFA 11+ Kids in soccer was associated with a reduction of the overall risk of injuries in children by 48%, and of 74% for serious injuries. However, to the best of our knowledge, a systematic review of the literature on the effects of FIFA 11+ Kids is still missing. Therefore, a systematic review was conducted to ascertain the benefits of the “FIFA 11+ KIDS” program in children who practice soccer.

**Methods:**

This systematic review was conducted according to the PRISMA recommendations and prospectively registered in PROSPERO. The electronic search was conducted in the following databases: Web of Science, PubMed, Medline via Ovid, EMBASE and SportDiscuss via EBSCO. Database searches were performed in January 2024. This review included studies that evaluated the effects of the “FIFA 11+ KIDS” program. Eligible studies had to describe program implementation and the mean age of the children.

**Results:**

A total of 11 articles were included in this systematic review from a pool of 8513 articles screened across various databases. These articles involved over 10,000 young participants from 8 countries, primarily aged 7–14 years, with the majority being soccer athletes. Study quality varied, with four categorized as high, four as good, and three as fair quality. Objectives varied across studies, with four focusing on FIFA 11+ Kids' efficacy in injury prevention, five examining its impact on performance and physical abilities, and two assessing its effects on children's focus and attention skills. Notably, injury prevention studies reported around a 50% reduction in overall injuries and nearly 60% in severe injuries, with a dose–response relationship observed with increased weekly sessions. Significant improvements were noted in physical and functional tests such as the Y balance, jump tests, and various soccer skills, along with positive effects on children's focus and attention, as indicated by 13–18% improvements in Attention Scale for Elementary School Children (ASESC) scores.

**Conclusion:**

The FIFA 11+ KIDS injury prevention program appears to be effective in reducing injuries in young football players. This can positively influence player and team overall performance and might support the long-term athlete development of these young athletes. These findings highlight the importance and necessity of injury prevention in young athletes.

## Introduction

Physical activity and a healthy lifestyle from a young age can promote a lifetime of healthy, active behaviour [7, 20, 30]. Soccer is one of the most commonly practised sports by young people around the world [8]. However, the epidemiology of injuries in children who practice soccer remains unclear [9, 28, 29]. particularly in the age group of 7 to 12 years, where injury patterns differ from those in adults. Preventive programs tailored to the type of sport and skeletal maturity of children are essential [15, 27, 28, 37] and the traditional “FIFA 11+ ”, also known as “11+ ” [13, 22, 32–34], is an injury prevention program designed for soccer athletes aged over 14, which has also been validated in other disciplines [1, 14, 17, 29, 32]. The FIFA 11+ Kids program focuses on (1) spatial orientation, anticipation, and attention, particularly during dual tasking to avoid unintentional contact with other players or objects; (2) body stability and movement coordination, which is more general than specific neuromuscular or proprioceptive training; and (3) learning proper fall techniques to minimize the consequences of unavoidable falls [26]. The use of FIFA 11+ Kids in soccer has been associated with a reduction in the overall risk of injuries in children by 48%, and 74% of serious injuries [27, 40, 42]. Therefore, a systematic review was conducted to identify the benefits and applications of the “FIFA 11+ KIDS” program in children.

## Material and methods

### Eligibility criteria

This review included studies that evaluated the effects of the “FIFA 11+ KIDS” program. Eligible studies described the implementation of the program and the mean age of the included children, with the maximum age being 14 years, regardless of gender. Only studies investigating the effect of the “FIFA 11+ KIDS” program in soccer were considered. Studies that did not report the duration or frequency of the program were excluded, as well as those that did not specifically mention the “FIFA 11+ KIDS” program. Only articles published in English, German, Portuguese, Spanish, French, and Italian were considered. No additional filters were applied to the database searches, and no time constraints were imposed. Grey literature, theses and dissertations were not considered.

### Search strategy

This systematic review was conducted following the Preferred Reporting Items for Systematic Reviews and Meta-Analyses (PRISMA) recommendations [19] and prospectively registered in PROSPERO (ID: CRD42022330012). The electronic search was performed across multiple databases, including Web of Science, PubMed, Medline via Ovid, EMBASE, and SportDiscuss via EBSCO, in January 2024. Various keywords were utilized in the search strategy, with adjustments made for each platform as necessary (see Table 1).

**Table 1:** Search strategy

Enquiry	Keyword
#1	Athletes
#2	Kids
#3	Child^a^
#4	Adolescen^a^
#5	Teen^a^
#6	Youth
#7	2 OR 3 OR 4 OR 5 OR 6
#8	1 AND 7
#9	Program
#10	Warm-up
#11	The 11
#12	FIFA
#13	FIFA 11
#14	FIFA 11+
#15	FIFA 11+ kids
#16	F-MARC
#17	9 OR 10 OR 11 OR 12 OR 13 OR 14 OR 15 OR 16
#18	Prevent^a^
#19	Injur^a^
#20	Risk reduction
#21	Intervention
#22	18 OR 19 OR 20 OR 21
#23	Not adult^a^
#24	8 AND 17 AND 22 AND 23

^a^Search strategy used in Medline (the strategy was modified according to the specifics of each database)

### Screening

Data extraction and study selection were independently conducted by two reviewers (**;**). The eligibility criteria were initially applied to exclude titles, followed by abstracts, and finally full articles. Additionally, the bibliography of the included studies underwent manual screening. In cases where consensus could not be reached between the two reviewers, a third reviewer (**) was consulted.

### Methodological quality and data synthesis

The methodological quality of the studies was assessed using the PEDro scale, which is based on the Delphi list [39, 44]. This scale comprises 11 questions, of which only 10 are scored, resulting in a scale ranging from 0 to 10. Each criterion is scored based on its presence or absence in the study being assessed. The final score is determined by summing all positive responses. The PEDro scale evaluates internal validity (items 2–9), statistics and results (items 10–11), and external validity (item 1).

A study is considered of high quality if it scores greater than 50% of its maximum possible score [24, 25, 38, 39]. However, given the inherent challenges in achieving certain conditions, such as blinding therapists or subjects in intervention studies, the maximum score for an intervention study should be around 80% [16]. Therefore, studies with a score greater than or equal to 8 were considered to have high methodological quality. Conversely, studies with a score of less than 3 were excluded from the present study because of their poor methodological quality.

## Results

### Study selection

**Figure 1:**
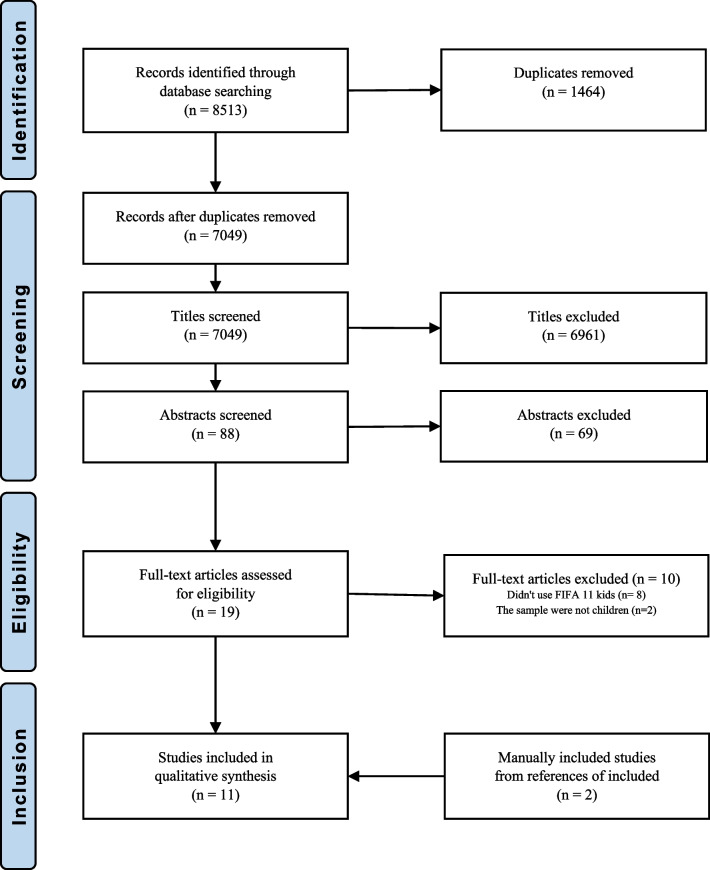
Flow chart of the search process

A total of 8513 articles were identified during the database search. After removing duplicates (N = 1464) screening titles (N = 6961) and abstracts (N = 79) that did not match the topic, a further 8504 articles were excluded. Nine articles met the inclusion criteria initially. Additionally, two articles were identified during the bibliography screening. Thus, a total of 11 articles were included in the literature search [3, 5, 23, 26, 27, 30, 35, 36, 41–43] (Fig. 1).

### Quality of included studies

Studies varied in quality, with scores ranging from 5 to 9 on the PEDro scale. Four studies were categorized as high-quality [23, 26, 35, 42], four studies as good quality (5 to 7), and three studies as fair quality (4). Most studies adequately reported baseline characteristics between groups, interventions, and results. However, the lack of blinding and allocation concealment were commonly reported limitations highlighted by the PEDro score (Table 2).

**Table 2:** Methodological quality assessment (PEDro score)

Author and year of publication	1	2	3	4	5	6	7	8	9	10	11	Total
Beaudouin et al. [3]	Yes	No	No	Yes	No	No	Yes	Yes	Yes	Yes	Yes	6
Chen et al. [5]	Yes	No	No	Yes	No	No	Yes	Yes	Yes	Yes	Yes	6
Pomares-Noguera et al. [23]	Yes	Yes	Yes	Yes	No	No	Yes	Yes	Yes	Yes	Yes	8
Rössler et al. [30]	No	No	No	Yes	No	No	No	Yes	Yes	Yes	Yes	5
Rössler et al. [26]	Yes	Yes	Yes	Yes	No	No	Yes	Yes	Yes	Yes	Yes	8
Rössler et al. [27]	Yes	Yes	Yes	Yes	No	No	No	Yes	Yes	Yes	Yes	7
Teixeira et al. [35]	Yes	Yes	Yes	Yes	No	Yes	Yes	Yes	Yes	Yes	Yes	9
Tseng et al. [36]	Yes	No	Yes	Yes	Yes	No	No	Yes	Yes	Yes	Yes	7
Zarei et al. [41]	Yes	No	No	Yes	No	No	No	Yes	Yes	Yes	Yes	5
Zarei et al. [43]	No	Yes	No	No	No	No	No	Yes	Yes	Yes	Yes	5
Zarei et al. [42]	Yes	Yes	Yes	Yes	Yes	Yes	No	Yes	Yes	Yes	Yes	9

1. Eligibility criteria were specified

2. Subjects were randomly allocated to groups

3. Allocation was concealed

4. The groups were similar at baseline regarding the most important prognostic indicators

5. There was blinding of all subjects

6. There was blinding of all therapists who administered the therapy

7. There was blinding of all assessors who measured at least one key outcome

8. Measures of at least one key outcome were obtained from more than 85% of the subjects initially allocated to groups

9. All subjects for whom outcome measures were available received the treatment or control condition as allocated or, where this was not the case, data for at least one key outcome was analyzed by “intention to treat”

10. The results of between-group statistical comparisons are reported for at least one key outcome

11. The study provides both point measures and measures of variability for at least one key outcome

### Characteristics of included studies

The extracted data including outcome measures associated with FIFA 11+ are summarized in Table 3. The analysis included more than 10,000 young participants from 8 different countries. The participants ranged in age from 7 to 14 years, with only 10% (107) not being athletes. All athletes included in the analysis were soccer players. Furthermore, four studies (36.3%) had a sample size exceeding 100 participants.

**Table 3:** Generalities and patient characteristics of the included studies

AUTHOR/YEAR	Location of the study	Sample	11+ protocol	Evaluation	Objective	Outcome
BEAUDOUIN et al. [3]	Czech Republic, Germany, Netherlands and Switzerland	Registered athletes from 7 to 12 years old (n: CON = 1829, INT = 2066)	The INT group performed '11+ Kids' 2x/week before training, and the CON group followed their regular training regimen for one season (2014/2015)	Information about practices, games, and injuries was recorded in a standardized online recording program.	To evaluate the effects of the '11+ Kids' injury prevention program in reducing serious injuries in 7- to 13-year-old football players	Overall serious injury incidence rates: 0.33 per 1000 h of football (95% CI 0.25 to 0.43) in CON and 0.15 (95% CI 0.10 to 0.23) in INT
Chen et al. [5]	Taiwan	52 children divided: trained boys (n = 13), trained girls (n = 13), control boys (n = 13) and control girls (n = 13)	Trained groups performed FIFA 11+ Kids for 8 weeks, 5x/week. Control groups were deprived of any exercise during the study period	The Chinese version of the Attention Scale for Elementary School Children (ASESC) test at baseline and one week after the intervention	To compare gender differences in attentional adaptation after an 8-week FIFA 11+ for Kids training intervention in elementary school children	Baseline vs post-training: total ASESC, focused attention, sustained attention, and selective attention. Trained boys: total ASESC scale, focused attention, sustained attention, selective attention and divided attention. Training girls: sustained attention and selective attention (*p* < 0.05)
Pomares-Noguera et al. [23]	Spain	23 young players (age: 11.8 ± 0.3 years) randomized into two groups (CON vs. INT)	The INT group performed FIFA 11+ Kids 2x/week for 4 weeks; the CON group completed normal warm-up routines	13 measures of physical performance, dynamic postural control, 20 m sprint time, ball slalom dribbling, agility, vertical jump height, horizontal jump distance, accuracy when throwing a ball were evaluated	To analyze the effects of FIFA 11+ kids training on different parameters of physical performance in male youth soccer players	Improvement in dynamic postural control ANT = 1 cm, PM = 5.1 cm and PL = 4.8 cm, agility run 0.5 s, vertical jump height = 3.1 cm and DJ = 1.7 cm and horizontal jump distance = 2.5 cm
Rössler et al. [30]	Switzerland, The Netherlands, Germany and the Czech Republic	Boys and Girls, ages 7–12, who played football in season (August-June)—614 (INT); 388 (CON)	The INT group used the ‘11+ Kids’ program 2X per week for 10 months and the CON control group performed their usual warm-up routine	Football-related injuries were recorded and followed up to 3 months after the end of the season Information about support and costs was obtained through telephone contact with the parents of injured children	To evaluate a potential reduction in injury related healthcare costs when using the’11+ Kids’ injury prevention programme compared with a usual warmup in children’s football	The difference in cost per 1000 h of exposure was CHF-240.66 (95% CI − 406.89, − 74.32). A nationwide roll-out would reduce healthcare costs in Switzerland by CHF 1.48 million per year
Rössler et al. [26]	North-Western Switzerland	157 children of the following age categories: sub-9, sub-11 and sub-13 years	INT conducted the 15-min “FIFA 11+ Kids” warm-up program 2X per week for 10 weeks and CON followed a standard warm-up. (INT, N = 56 players and CON, N = 67)	Single leg stance; Y-balance test; drop and countermovement jump; standing long jump; 20-m sprint; agility run; slalom dribble; and wall volley test	Evaluate the effects of FIFA 11+ Kids on the motor performance of children aged 7 to 12 years	There were significant effects Y balance (right leg; + 3.2%; SMD = 0.34; *p* = 0.58) and agility running (+ 3.6%; SMD = 0.45; *P* = 0.008)
Rössler et al. [27]	Czech Republic, the southwest of Germany, the Netherlands and the German-speaking cantons of Switzerland	3895 players (Under 9 to Under 13) CON = 1829 players; INT = 2066 players	The INT group did '11+ Kids' 2X per week for 10 months at the beginning of their training sessions, while the CON group followed their regular training	The exposure time and injuries suffered were collected using an injury registration system	Evaluate the effectiveness of 11+ Kids to reduce injuries in children's soccer	The overall injury rate in the INT group was reduced by 48%. Severe injuries were reduced by 74% and lower extremity injuries reduced by 55%
Teixeira et al. [35]	Brazil	24 male youth soccer athletes (9–11 years)	8 weeks, with three training sessions per week, totaling 24 training sessions in the INT group n = 18, while the CONT group performed conventional training	Kinetic assessments of vertical jump (VJ), drop landing (DL), and anterior jump + maximum vertical jump (AJ)	To evaluate the FIFA 11+ Kids effects on jumping kinetics in youth soccer player	Post-intervention impulse peak force and maximum impulse force (VJ), Landing peak force values for the first and second landings (DL) and Landing peak force in the first landing (AJ) were significantly greater in the INT group
Tseng et al. [36]	Taiwan	55 elementary school students; INT (n = 28), CON (n = 27)	the INT group did FIFA 11+ kid 5 times a week for 8 weeks. In addition, children in both groups were asked to maintain their usual physical activities and physical education classes (two 50-min classes per week) during the study period	The following were evaluated: (1) sit and reach, (2) long jump, (3) sit test, and (4) 800 m run test In addition, tests of focused attention, sustained attention, selective attention, alternating attention and divided attention were performed	To investigate the effects of 8-week FIFA 11+ kid intervention on physical fitness and attentional capacity in elementary school children	The INT group improved on sit and reach (10.7%); long jump (2.44%); sit(12.90%); and 800 m running (− 1.92%) In the Attentional Assessment, total, focused, selective scores; and the alternating attentions in the INT group were significantly higher
Zarei et al. [41]	Iran	31 football players from 2 teams between 10 and 12 years of age. CON (n = 15), INT (n = 16)	The INT group used the “11+ kids” program for 10 weeks as a warm-up at the start of their training sessions. The control group followed their usual warm-up program	Isokinetic strength of the hip adductors and abductors, knee flexors and extensors, and ankle invertors and evertors was tested	to investigate the effect of 11+ kids compared to a traditional warm-up for 10 weeks Isokinetic strength parameters of the muscles around the hip, knee and ankle in young soccer players were evaluated	The INT group had an increase in isokinetic strength of hip adductors and adductors (10%) (9.8%), knee flexors (6.7%), ankle evertors (12.1%) and inverters (9, 8%)
Zarei et al. [43]	Spain	42 adolescent soccer players (9–14 years old) (INT = 19; CON = 23)	The INT group did the 11+ kids for 10 weeks and the CONT followed the in-season warm-up routine	To measure the performance and the physical fitness, we used dribbling, Illinois agility, flexibility, standing high jump, triple hop, Y balance, 20 and 40-yard speed, plank, and side plank tests	To analyze the effects of FIFA 11+ kids training on physical performance in young soccer players	There was significant improvement between groups for the 40-yard sprint test (*P* = 0.002), the Y-balance test in the posterior (*P* = 0.001), medial (*P* = 0.001) and lateral (*P* = 0.001) and also the triple hop test (*P* = 0.002)
Zarei et al. [42]	Iran	962 players (CON: 519 players, INT: 443 players) between 7 and 14 years old	The INT group used 11+ Kids at least twice a week replacing the usual warm-up. The CON group maintained the standard warm-up	Exposure time and information about the use of 11+ Kids was entered by trainers after each training using electronic forms	To evaluate the effectiveness of 11+ Kids in reducing injuries in high-level male children's soccer players	The incidence of injury in the INT group was reduced by 50% compared to CON (RR 0.50; 95%-CI 0.32, 0.78). There were no injuries during the execution of the intervention exercises

### Objectives

Four studies verified the efficacy of FIFA 11+ Kids in preventing injuries in young soccer athletes [3, 27, 30, 42], while five studies focused on improving athletes' performance and physical abilities [23, 26, 35, 41, 43]. One study analyzed the effects of FIFA 11+ Kids on attention, focus, and physical skills [36], and another study investigated whether FIFA 11+ Kids improved attention and focus skills [5]. Injury prevention studies utilized face-to-face or online monitoring methods to track injury incidence, with data often provided directly by team coaches. Among the physical and functional tests employed, the Y balance, jumping tests, and assessments of skills such as dribbling and agility were most commonly utilized.

### Injury rate

Four studies (with a total of 8752 young athletes) evaluated the injury index as the main outcome [3, 27, 30, 42]. These studies observed a reduction rate of approximately 50% in injuries resulting from sports practice and almost 60% in the incidence of severe injuries (defined as time away from work > 28 days), with values of 0.13 injuries per 1,000 h of exposure in the experimental group and 0.31/1,000 h in the control group [27, 42, 43]. Furthermore, a greater number of weekly sessions contributed to a greater reduction in the incidence of injuries, with evidence of a dose–response relationship [4, 27, 42, 43].

### Performance

Five studies (involving 277 young athletes) assessed performance and physical abilities as the main outcomes [23, 26, 35, 41, 43]. The studies collectively show the FIFA 11+ Kids program positively affects various aspects of physical performance in young football players. Tests like Y balance, jump tests, dribbling, and agility consistently improved following the interventions. For example, Pomares-Noguera et al. [23] observed enhancements in dynamic postural control, agility, vertical and horizontal jump height, and accuracy in ball throwing among male youth soccer players after implementing the program. Similarly, Rössler et al. [26] noted significant improvements in motor performance, especially in Y balance and agility running tests, in children aged 7 to 12 years participating in the program.

### Focus and attention

Two studies analysed the effects of FIFA 11+ KIDS on the focus and attention of children [5, 36]. The total score of the Attention Scale for Elementary School Children (ASESC) improved by 13% to 18%.

## Discussion

The FIFA 11+ injury prevention program for kids primarily targets improvements in coordination and balance, strengthening of leg muscles, and optimization of landing techniques. As demonstrated in this study, FIFA 11+ KIDS proved to be significantly more effective than control programs in reducing injury rates, enhancing performance, and improving focus and attention.

Recent findings from a systematic review and meta-analysis revealed that FIFA 11+ Kids significantly reduces injury risks among young football players [40]. This analysis, incorporating six studies from January 2016 to August 2022, demonstrated a notable decrease in overall injury risk, severe injuries, as well as specific injuries to the lower extremities, knees, and ankles. These results provide robust support for the widespread implementation of FIFA 11+ Kids as an effective injury prevention strategy in youth football.

Our study aimed to investigate additional performance and mental health variables beyond injury rates, showcasing the broader impact of the FIFA 11+ KIDS program. This intuitive program requires only 20 min to complete, progressing through 5 stages of increasing difficulty with 7 exercises each, accommodating varying levels of motor skill development [31]. Initially designed for youth soccer injury prevention, FIFA 11+ KIDS has proven adaptable to diverse populations, including non-athletic children [10]. Moreover, it has shown effectiveness in enhancing various outcomes, including performance, focus, and attention [6, 12, 36].

Enhancing children's focus and attention through interventions such as the FIFA 11+ KIDS program can lead to improved learning outcomes and academic performance, as these cognitive functions are integral to academic success [12]. Educational administrators and teaching staff in elementary schools must consider implementing physical activity interventions, such as the FIFA 11+ KIDS program, to enhance the fitness levels and overall well-being of school children.

The implementation of the programme in schools can be a challenge, as the intensity and duration of the training intervention might not be feasible in many settings [18]. Adjustments to fit the needs of schools are recommended, and modifying the frequency and duration of the FIFA 11+ KIDS or changing the schedule of exercise interventions could be considered [10].

The FIFA 11+ KIDS program has demonstrated remarkable effectiveness in preventing football injuries, surpassing conventional warm-up routines by reducing overall injuries by at least 50% and severe injuries by almost 60%. Similar assessments of the FIFA 11 and FIFA 11+ programs in adult football players have shown a significant reduction in injury risk, with football injuries decreasing by 39% [2, 31, 36]. These findings underscore the substantial impact of FIFA's injury prevention initiatives in mitigating injury rates in both adult and child football players.

Decreasing injury incidence not only enhances performance throughout a sporting season but also fosters improved athletic development among young athletes [11]. Moreover, injury reduction initiatives such as the 11+ KIDS program can yield long-term benefits. One factor that may hinder the program's effectiveness is the low adherence among young athletes and coaches. Higher completion rates of the FIFA 11+ KIDS program are associated with greater protective effects [2]. Coaches may exhibit resistance to change, opting to stick to familiar routines or traditional warm-up exercises. Similarly, athlete non-compliance can lead to suboptimal execution of the program or high dropout rates [21].

The inclusion of studies with varied outcome measures limited the direct comparison of intensities and efficacy of protocols across studies. Statistical analysis, such as examining the relationship between outcomes, was beyond the scope of this systematic review. However, such analyses could offer valuable insights into optimizing the prescription of the FIFA 11+ KIDS protocol. Future studies could explore the transferability of the FIFA 11+ KIDS program to other team sports.

### Limitations

While the FIFA 11+ Kids program holds promise to improve injury prevention in young athletes, its effectiveness remains less clear compared to the well-established adult FIFA 11+ program. The current body of research is limited, particularly regarding adherence rates, which can vary depending on factors like the specific program format, study population characteristics, and methodological approaches. Some studies report high adherence, while others show lower rates because of lack of motivation or logistical challenges. Analysing adherence data from each study would provide a precise range, but unfortunately, these data are missing from the reviewed studies. This lack of data hinders our ability to draw definitive conclusions about the program's long-term impact on injury reduction and its suitability for different age groups, genders, and skill levels. Future investigations should explore the implementation of FIFA 11+ Kids in broader demographics, accounting for factors such as athlete characteristics and training environments. Additionally, employing robust methodologies that capture adherence data will be crucial for establishing the program's effectiveness and identifying optimal use guidelines.

## Conclusion

The FIFA 11+ KIDS injury prevention programme demonstrates effectiveness in reducing injuries in young football players, potentially enhancing both individual player and team performance. These outcomes underscore the significance of injury prevention initiatives in young athletes, particularly those engaged in high-performance sports, and emphasize the importance of implementing such programmes for long-term athlete development.

## Data Availability

The data underlying this article are available within the article.
